# Evaluation of 17β-Estradiol Administration by Subcutaneous Injections in Transgender Women

**DOI:** 10.1210/jendso/bvaf192

**Published:** 2025-11-25

**Authors:** Hadley M Moreau, Haley Perkins, Jennifer LaBudde, Wendy Y Craig, Daniel I Spratt

**Affiliations:** Tufts University School of Medicine, Portland, ME 04102, USA; Department of Obstetrics and Gynecology, MaineHealth Maine Medical Center, Portland, ME 04102, USA; Department of Surgery, UMass Chan Medical School, Worcester, MA 01655, USA; MaineHealth, Memorial Hospital, Conway, NH 03813, USA; Tufts University School of Medicine, Portland, ME 04102, USA; MaineHealth Institute for Research, Portland, ME 04102, USA; Tufts University School of Medicine, Portland, ME 04102, USA; Department of Obstetrics and Gynecology, MaineHealth Maine Medical Center, Portland, ME 04102, USA

**Keywords:** transgender, estradiol, subcutaneous, testosterone

## Abstract

**Context:**

17β-Estradiol (E2) is increasingly administered subcutaneously (SC) to transgender women, but questions remain regarding dosing and efficacy.

**Objectives:**

The primary aims of this work were to determine if SC E2 could consistently achieve serum E2 and total testosterone (TT) levels within target therapeutic ranges and if TT and E2 levels were comparable to those achieved with standard oral therapy. The secondary aim was to determine if serum estrone (E1) and sex hormone–binding globulin (SHBG) levels were lower with SC compared to oral administration, possibly reflecting fewer first-pass hepatic effects.

**Methods:**

This retrospective cohort study evaluated records of transgender women receiving either SC or oral E2 in the Reproductive Endocrinology and Gender Clinics at Maine Medical Center. Serum levels of E2, TT, E1, and SHBG were extracted from charts.

**Results:**

Demographics were similar in SC (n = 25) and oral (n = 20) groups. Serum E2 reached the target therapeutic range (75-250 pg/mL) and TT was suppressed to less than 50 ng/mL in all patients and were not statistically different between groups. In 5 patients in the SC group, E2 was measured before and after dosing, with mean values within the target range. The median (interquartile range) E1 level was higher in the oral E2 group than in the SC E2 group (907 pg/mL [737-1576 pg/mL] and 76 pg/mL [49-96 pg/mL]; *P* < .001). SHBG levels did not differ between groups.

**Conclusion:**

SC E2 administration is effective in transgender women for achieving target serum E2 and TT levels. Both SC and orally administered E2 achieved similar E2 and TT levels, but orally administered E2 resulted in much higher E1 levels.

17β-Estradiol (E2) therapy is commonly prescribed to postmenopausal, hypogonadal, and transgender women. E2 is the standard of care to achieve feminization in gender-affirming hormone therapy (GAHT) and is typically used in higher doses in the transgender population than in postmenopausal women. E2 is administered orally, transdermally, and, less frequently, intramuscularly (IM). Recently, subcutaneous (SC) injection of E2 has gained popularity among transgender women and is being used for GAHT at increasing rates, despite a paucity of data regarding its efficacy and safety [1]. To our knowledge, there are only 2 studies reporting serum E2 and total testosterone (TT) levels for adult transgender patients using SC E2. A study by Herndon et al [2] included 74 patients on SC therapy (64 on valerate, 10 on cypionate) and reported that more than half of the participants had serum E2 levels outside the accepted therapeutic range of 100 to 200 pg/mL with a median serum E2 value of 196 pg/mL. Despite these data indicating that half the serum E2 levels were above 196 pg/mL, 13.2% of these patients did not have serum TT levels suppressed to within the normal female range (<50 ng/dL) [2]. A recent multicenter study evaluating SC and IM administration of E2 reported that of the patients who had serum E2 levels between 100 and 200 pg/mL and no orchiectomy or gonadotropin-releasing hormone (GnRH) agonist (GnRHa) or antiandrogen therapy, only 21.6% (19/88) achieved serum TT less than 50 ng/dL [3]. In a reanalysis of these data, Misakian et al [4] reported that among patients without a history of gonadectomy or concurrent GnRHa use, 12 out of 90 patients treated with SC E2 failed to achieve serum TT concentrations below 50 ng/dL despite many having serum E2 concentrations greater than 200 pg/mL [4]. Taken together, these studies raise concern regarding the efficacy of SC E2 in GAHT with respect to achieving target ranges of serum TT concentrations with serum E2 concentrations within the target therapeutic range. Furthermore, these reports did not include information regarding safety or effect of first-pass hepatic effects.

Current guidelines from the Endocrine Society and the World Professional Association for Transgender Health (WPATH) recommend E2 via oral, transdermal, or IM routes, with a combination of patient preference and patient risk factors generally guiding route selection [5, 6]. Each mode of administration has unique limitations. Oral E2 is generally taken once or twice daily, which some patients find cumbersome. Additionally, oral E2 has been associated with an increased risk of venous thromboembolism (VTE) when compared to transdermal E2, which is thought to be partly due to first-pass hepatic metabolism and amplification of thrombotic factors [7-10]. Studies suggest that transdermal E2 is associated with lower VTE risk than oral therapy both in transgender and postmenopausal women, but it is associated with increased cost, adhesion issues, local allergic reactions, and overall decreased patient acceptance [9, 11-14]. Patients who prefer IM E2 often cite the convenience of once-weekly or biweekly dosing, but IM injections are associated with a larger bore needle, which some patients find painful. Potential benefits of SC E2 include the convenience of self-administered weekly injections in addition to a smaller-bore needle than used for IM injection. In a study on transgender men, SC T therapy was preferred over IM for many of these reasons [15].

Our study had 5 objectives. First was to determine if in a single clinic, carefully adjusted doses of SC E2 could reliably achieve therapeutic serum E2 concentrations and suppress serum TT concentrations to less than 50 ng/dL. Second was to compare the serum E2 and TT concentrations attained with SC E2 therapy with those attained with the more commonly used oral therapy. Third was to obtain preliminary evidence assessing variations in serum E2 concentrations between weekly dosing intervals. Fourth was to evaluate safety of SC injections with respect only to immediate local and systemic reactions. The final objective was to obtain preliminary evidence for differences in first-pass hepatic metabolism between SC and oral administration by comparing serum levels of estrone (E1) and sex hormone–binding globulin (SHBG). There remains a paucity of data regarding the safety risks associated with the relatively high doses of E2 used in GAHT for transgender women, with some evidence suggesting that first-pass hepatic products such as E1 may contribute to an increased risk of VTE [10]. Although safety is not directly evaluated in this study, serum E1 was routinely monitored at least once after optimizing the E2 dose in our clinical practice to assess if E1 levels were elevated and to what extent.

## Materials and Methods

### Participants and 17β-Estradiol Dosing

Patients in the MaineHealth Gender and Reproductive Endocrinology Clinics in Portland, Maine, undergoing male-to-female transition between the dates of May 30, 2014 and May 30, 2023, taking either oral E2 or SC E2 valerate therapy and who were routinely monitored as a part of standard clinical practice were potentially eligible for inclusion. Assignment of oral, transdermal, or SC E2 was according to patient preference and safety parameters, if relevant. Patients were included who had completed an individualized dose-adjustment process of escalating doses of E2 (with limits preset at maximum doses of 10 mg/week SC or 12 mg/day orally). If a patient were to reach these upper limits of dosing but not reach the target therapeutic range of E2 75 to 250 pg/mL, this would be assessed as “treatment failure.” Endocrine Society guidelines suggest a target range of 100 to 200 pg/mL [5]. If our patients had serum E2 levels between 75 and 100 pg/mL with a serum T level suppressed to within the target range of less than 50 ng/dL suggested by Endocrine Society guidelines and were achieving their clinical goals, the E2 dose was not further increased [5]. If our patients had serum E2 levels between 100 and 200 pg/mL and serum T was not yet suppressed to less than 50 ng/dL, the E2 dose was increased to achieve a serum E2 level up to 250 pg/mL. If the serum E2 were to reach 250 ng/dL but the serum T was still greater than 50 ng/dL, this was also assessed as “treatment failure.” All 45 patients included in the study completed the dose adjustment period and met the following additional criteria for inclusion: (1) age 18 to 79 years, (2) had been taking the same dose of E2 for at least 30 days, and (3) no concomitant GnRHa therapy. This retrospective cohort study was reviewed as exempt by the MaineHealth Institutional Review Board. Informed consent was not required.

### Administration of 17β-Estradiol

Patients were prescribed E2 doses beginning at 2 mg/day orally or 2 mg/week SC and adjusted as described earlier with dose adjustments targeted at achieving serum E2 of 75 to 250 pg/mL and T less than 50 ng/dL. If patients were to reach doses of 12 mg/day orally or 10 mg/week without obtaining a serum E2 level greater than 75 pg/mL and serum TT less than 50 ng/dL, then this would be judged as treatment failure in terms of these 2 parameters of efficacy. Those on oral E2 therapy were prescribed generic E2 and instructed to swallow their dose either once or twice daily. If a patient was prescribed 2 mg orally, E2 was dosed once daily. At any dose above 2 mg, patient preference and the ability to comply directed once vs twice daily dosing. Patients on SC therapy were prescribed E2 valerate (10 mg/mL) due to the increased availability of valerate over cypionate in local pharmacies, in addition to the lower viscosity and cost of this preparation. Prior to initiation of SC E2, patients received educational materials and were observed performing the initial injection in the office. Patients were taught to draw a dose of E2 valerate in a 1-mL Luer-Lok syringe and administer the medication at approximately a 45° angle under the skin of the abdomen or thigh using a 25-gauge 5/8-inch needle. The Luer-Lok syringe was used to prevent the needle from separating from the syringe during injection due to the viscous nature of the E2 valerate solution. After the initial observed injection, all patients performed their own SC injections once weekly.

### Administration of Progestogen or Spironolactone

Spironolactone therapy (100-200 mg daily) was offered to all patients at the same time as E2 therapy, due to its potential to reduce facial and body hair. Although spironolactone may decrease serum TT levels, it was not prescribed for that reason. Micronized progesterone or medroxyprogesterone therapy were offered to patients to possibly enhance breast development if desired after the E2 dose was stabilized.

### Monitoring

While receiving SC E2, serum levels of E2 and TT were measured 1 to 2 days after each patient's fourth injection on a given SC dose. For a subset of patients, serum E2 levels were also measured within 24 hours prior to an injection (6 days following the previous dose). Pre and post measurements were obtained within the same 30-day period. Monitoring of hormone levels in those taking oral E2 were not timed in relation to time from dose. Markers of first-pass hepatic effect (SHBG and E1) were routinely monitored at least once on an optimized E2 dose in our clinical practice. For the study, we include only serum E1 and SHBG measurements that were obtained within 3 months of a serum E2 measurement, while patients were on a stable and consistent dose of E2. Patients were asked at each office or telehealth visit about breast tissue development, body fat redistribution, changes in skin texture, changes in muscle mass or strength, and changes in body hair. Additionally, patients were evaluated for local injection site reactions and symptoms of immediate systemic or allergic response through history and physical examination.

### Serum Hormone Assays

Serum total E2, TT, and total E1 were measured via liquid chromatography/mass spectrometry (LabCorp). Proficiency tests for these assays were concordant with the Centers for Disease Control and Prevention reference method assays. Interassay precision for low, medium, and high concentration quality control serum samples, respectively, expressed as percentage coefficient of variation were: T, 9.9%, 7.9%, and 5.0% and E2, 4.4%, 3.5%, and 3.3%. SHBG levels were measured via a 2-step immunoenzymatic assay (Mayo Clinic Laboratories) with a normal adult premenopausal female range of 18.2 to 135.5 nmol/L.

### Data Analysis

Demographic and clinical data were reported as a mean ± SD (full range), mean (range), or n (%) as indicated. Serum hormone measurements of E2, T, SHBG, and E1 are shown as median (interquartile range, IQR) (full range) or mean (95% CI) (full range) as indicated. Differences in continuous variables between subgroups were evaluated either by *t* test or Mann-Whitney *U* test as appropriate. Local or systemic reactions were reported as n (%). All analyses were performed using SPSS Statistical Software version 29 (IBM SPSS Inc).

## Results

### Participant Characteristics

Characteristics of the 45 study participants are displayed in Table 1, stratified by E2 administration route. The study groups included 20 participants taking oral E2 and 25 taking SC E2 therapy. All patients were transgender women and none were nonbinary patients. All participants were residents of the state of Maine who identified as White and non-Hispanic. Other demographic and characteristics were similar between the oral and SC subgroups. The duration of follow-up of patients on the optimized E2 dose ranged from 4 weeks to 5 months.

**Table 1.** Demographic, clinical, and laboratory characteristics of the participants

Variable*^a^*	Route of E2 administration		
	PO	SC	*P*
n	20	25	
**Demographic and clinical characteristics**			
Age, y	36.7 ± 14.5	38.1 ± 17.1	—
Body mass index	26.4 ± 6.6 (n = 13)	27.9 ± 7.0 (n = 14)	—
Progestogen*^b^*	9 (45.0)	10 (40.0)	—
Spironolactone*^c^*	17 (85.0)	14 (56.0)	—
Progestogen + spironolactone	9 (45.0)	7 (28.0)	—
Prior orchiectomy	2 (10.0)	8 (32.0)	—
**E2 dosing and laboratory results**			
E2 dose, mg/wk	42 (29.7-42) (28-84)	3.4 (2.8-4.0) (1.4-6.0)	—
Serum E2, pg/mL*^d^*	151.6 (136.8-166.4)	147.3 (130.4-164.1)	.69*^g^*
Serum TT, ng/dL*^e,f^*	20.6 (8.2-36.2) (3.4-42)	14.0 (10.8-27.2) (2.5-39.7)	.44*^h^*
n	17	12	—
Serum TT, no progestogen	21.3 (9.2-37.4)	13.0 (8.8-29.7)	—
n	10	5	—
Serum TT, progestogen	18.0 (6.2-35.5)	14.0 (13.0-29.7)	—
n	7	7	—
First-pass serum hormone measurements			
E1, pg/mL	907 (737-1576) (596-1601)	76 (49-96) (39-115)	<.001*^h^*
n	5	11	
E1/E2 ratio	8.0 (4.3-11.6)	0.46 (0.43-0.54)	<0.001*^g^*
n	5	11	
SHBG, nmol/L	73.4 (59-116) (51-187.4)	72.4 (41.2-102.8) (19.3-146.7)	0.24*^h^*
n	14	16	

Abbreviations: E1, estrone; E2, estradiol; PO, standard oral; SC, subcutaneously administered; SHBG, sex hormone–binding globulin; TT, total testosterone.

^a^Categorical data shown as n (%); age and body mass index shown as mean ± SD; E2 dose shown as median (interquartile range) (full range); serum E2 level and E1/E2 ratio shown as mean (95% CI); TT, E1, and SHBG shown as median (interquartile range) (full range).

^b^In the PO group 6 patients were taking medroxyprogesterone (2.5-10 mg/d) and 3 were taking micronized progesterone (100-200 mg/d). In the SC group 2 patients were taking medroxyprogesterone (5-10 mg/d), and 8 were taking micronized progesterone (100-200 mg/d).

^c^Spironolactone dose range for patients in the PO group was 100 to 200/mg daily. The dose range for patients on SC therapy was 100 to 300 mg/daily.

^d^Adult female reference range for serum E2 is 75 to 250 pg/mL.

^e^Adult female reference range for serum TT is 10 to 55 ng/mL.

^f^TT data shown for participants without prior orchiectomy.

^g^ *T* test.

^h^Mann-Whitney *U* test.

### Dosing and Efficacy

Following 1 session injecting E2 under supervision in the office, all patients were able to successfully and without difficulty self-administer SC E2 at home. Doses used are displayed in Table 1. Median weekly E2 dose was 42 mg (range, 28-84 mg) for those on oral E2 and 3.4 mg (range, 1.4-6.0 mg) for those on SC E2.

**Figure 1.**
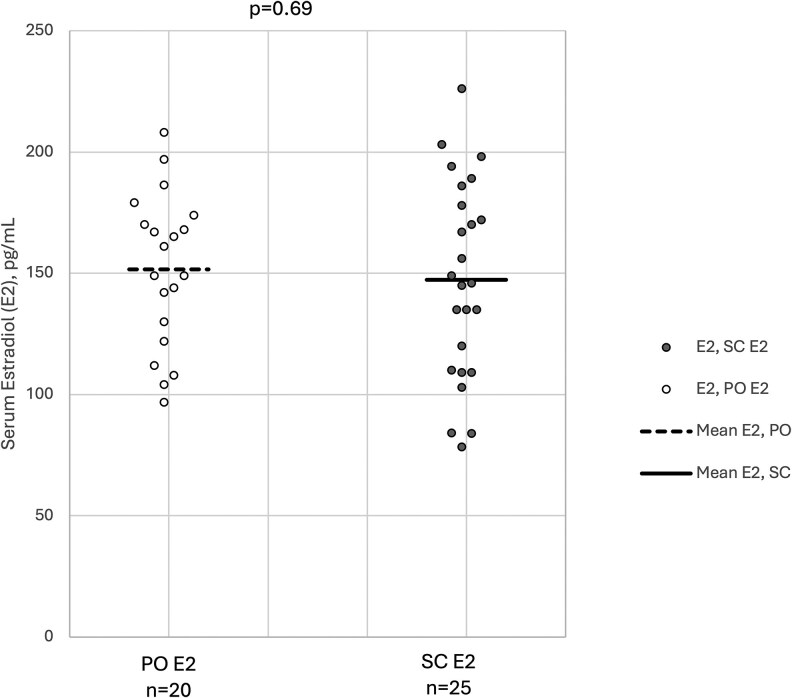
Serum estradiol (E2) measurements among participants, stratified by route of estradiol delivery. Serum E2 levels were available for 20 patients receiving standard oral (PO) E2 (left) and 25 patients receiving subcutaneous (SC) E2 (right). Bars indicate mean E2 serum level. Open (PO) and closed (SC) circles represent individual measurements.

**Figure 2.**
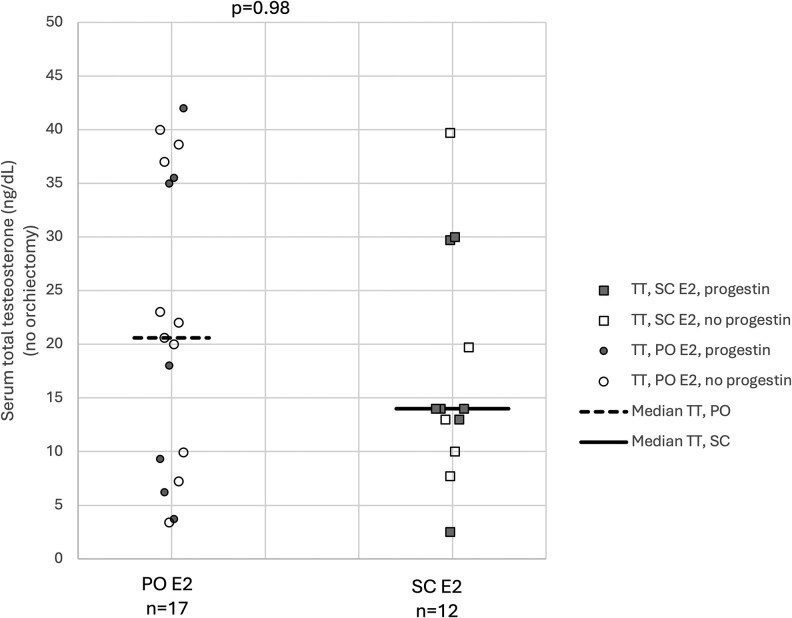
Serum total testosterone (TT) measurements among participants, stratified by route of estradiol (E2) administration. Participants with prior orchiectomy were excluded. Bars represent median values. Circles represent standard oral (PO) E2 delivery and squares represent subcutaneous (SC) E2 delivery. Open symbols indicate that the individual was not taking a progestogen, and closed symbols indicate that the individual was taking a progestogen.

Serum E2 and TT measurements are summarized in Table 1 and illustrated in Fig. 1 (E2) and Fig. 2 (TT). In both the SC E2 and oral E2 groups, all participants demonstrated serum E2 levels within the target therapeutic range (75-250 pg/mL). Of the 17 individuals without prior orchiectomy in the SC E2 group, 12 had data for TT and all were suppressed (TT <50 ng/dL). Of the 18 individuals without prior orchiectomy in the oral E2 group, serum TT data were available for 17 and all values were less than 50 ng/dL. There were no statistically significant differences in serum E2 between the oral and SC groups (mean [95% CI, 151.6 pg/mL [136.8-166.4 pg/mL] and 147.3 pg/mL [130.4-164.1 pg/mL]; *P* = .69) or in median (IQR) serum TT levels among those without prior orchiectomy (20.6 ng/dL [8.2-36.2 ng/dL] and 14.0 ng/dL [10.8-27.2 ng/dL]; *P* = .44).

**Figure 3.**
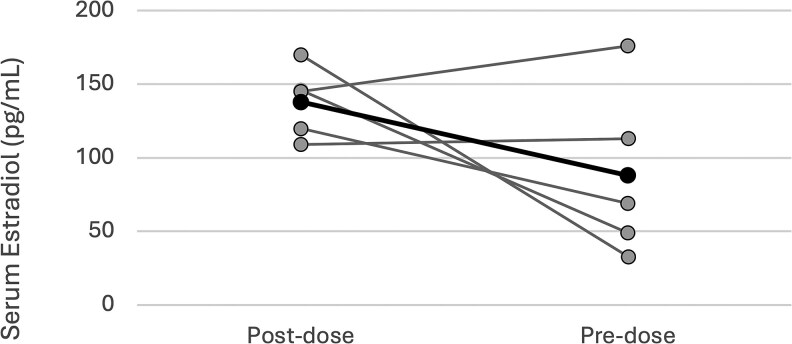
Serum estradiol (E2) levels among a subgroup of participants on subcutaneous (SC) E2 therapy with serum sampling 1 day prior to (pre dose) and 1 to 2 days following (post dose) E2 injection. Closed gray circles represent post- and pre-dose E2 levels for individual participants; the closed black circles represent median values. Two patients had stable serum E2 levels between injections, while 3 had a decline in values.

Fig. 3 displays post- and pre-dose E2 levels for the 5 individuals in the SC group with paired data. Post-dose is defined as a measurement taken 24 to 48 hours after a dose of SC E2 and pre-dose is defined as a measurement taken within 24 hours before an SC E2 injection. The mean (95% CI) post-dose E2 level was 138 pg/mL (108-168 pg/mL), and the pre-dose level was 88.0 pg/mL (16-159 pg/mL).

### Local and Allergic Reactions

Assessment of safety was limited to evaluation of local reactions through history and physical examination, as well as identification of immediate allergic or inflammatory systemic reactions based on reported symptoms. In the SC group, one participant (4.0%) reported a transient local reaction of swelling, redness, and tenderness at an injection site lasting 3 days following an injection. On physical examination, an area of mild induration surrounding the injection site was observed measuring approximately 2 × 4 cm that lacked erythema, warmth, or tenderness. The reaction resolved without intervention and the patient elected to continue with SC therapy and did not experience further local reaction. No other individuals had historical or physical evidence of a local reaction. No symptoms of systemic allergic or inflammatory reactions occurred following initiation of therapy in either the SC or the oral group.

### First-Pass Hepatic Effect

We measured serum levels of E1 and SHBG as markers for first-pass effect (see Table 1). The median (IQR) E1 level was significantly higher in the oral E2 group than in the SC E2 group (907 pg/mL [737-1576 pg/mL] and 76 pg/mL [49-96 pg/mL]; *P* < .001). This difference was further illustrated by a mean E1:E2 ratio of 8.0 (95% CI, 4.3-11.6) in the oral group and 0.46 (95% CI, 0.43-0.54) in the SC group. There was no statistically significant difference in median (IQR) serum SHBG levels between the oral E2 and SC E2 groups (73.4 [59-116] and 72.4 [41.2-102.8] nmol/L, respectively; *P* = .24).

## Discussion

Our study demonstrates that SC injection of E2 is an effective form of GAHT for transgender women with respect to achieving serum E2 and TT concentrations within our target therapeutic ranges. Following an adequate dose-adjustment period and at least 30 days on a consistent E2 dose, all 25 patients on SC therapy achieved serum E2 levels within our target range of 75 to 250 pg/mL that were comparable to levels observed in the group receiving standard oral therapy. Furthermore, suppression of serum TT to within the normal female range occurred in all patients in the SC group and was comparable to patients in the oral group. These results were achieved with SC E2 doses ranging from 1.4 to 6 mg per week.

Many of our patients, as in the recent multicenter study, were receiving concomitant progestogens and/or spironolactone [3]. Progestogens can contribute to central suppression of the hypothalamic-pituitary-gonadal axis and may have contributed to effective suppression of serum TT in these patients. Some studies have reported a decrease in serum TT levels with spironolactone use while others have not. Thus, spironolactone administration may also have contributed to decreased serum TT in some of our patients. An equal proportion of patients in our SC and oral patient groups were receiving a progestogen or spironolactone. Thus, overall, SC E2 administration appears as effective as oral administration in suppressing serum TT to less than 50 ng/dL, although it is possible that a progestogen or spironolactone may be needed to assist in this suppression.

Prior studies describing SC injection of E2 in transgender women raised concern for the efficacy of this therapeutic modality as these studies reported serum TT levels above the normal female range as discussed at the beginning of this work. Despite a large number of patients having serum E2 levels above the recommended target range of 100 to 200 pg/mL, a substantial number of patients did not have serum TT suppressed to less than 50 ng/dL [2-4].

With respect to SC E2 dosing, in a review article, Rothman et al [1] suggest that administering up to 10 mg/week of E2 cypionate or valerate via SC or IM injection may lead to supraphysiologic serum E2 levels exceeding the target therapeutic range. Consequently, they recommended starting doses of less than 5 mg/week both for E2 cypionate and valerate when administered parenterally [16]. Data supporting this notion were recently reported from a multicenter study [3]. As noted earlier, patients in our study achieved therapeutic E2 serum levels at SC doses ranging from 1.4 to 6 mg E2 per week and thus our data are congruent with Rothman's suggestion and the multicenter study that doses above 6 mg per week may produce serum E2 levels higher than the target therapeutic range in most patients. We found that using a starting dose of 2 mg weekly, we were able to achieve E2 and TT levels at goal in all patients after dose adjustment. Thus, this is a reasonable recommendation for other clinicians.

In this study we collected preliminary data investigating the stability of serum E2 levels between injections by evaluating serum E2 levels within 24 hours prior to as well as 1 to 2 days following SC injection. Though the mean serum E2 level was within the target range before and after SC injection, 3 out of 5 patients had an evident decline in E2 levels prior to the next injection and 2 patients had E2 levels below the target range 1 week following injection. These data are consistent with the Misakian study [3], which demonstrated that E2 levels tended to decline with increasing time since injection. Specifically, their study reported on average a 19-pg/mL decline in serum E2 level with each additional day since E2 injection. Although pharmacokinetic data are lacking with SC administration of E2, E2 valerate has been shown to have a peak concentration 24 to 48 hours following IM administration in healthy postmenopausal cisgender women [17]. Given these data, obtaining serum E2 measurements 1 to 2 days after an injection, as was our practice, is reasonable to assess dosing by peak serum E2 levels. Further evaluation of pharmacokinetics following SC injection will be helpful to determine if the optimum time for sampling is peak, mid-cycle, or trough.

Other studies describing SC E2 injection have not commented on local reactions or systemic inflammatory or allergic reactions [2, 3] In our study, no patients on SC E2 reported any symptoms of systemic reactions following initiation of therapy. Only one participant reported a transient injection site reaction that self-resolved with supportive care. While our data that were limited to adverse local and systemic reactions assessed by history and physical examination reflect this aspect of safety and a favorable tolerability profile, additional studies are needed to more fully assess other aspects of long-term safety of SC E2. It is noteworthy that the STRONG study assessed the incidence of VTE and ischemic stroke (IS) with E2 administration to transgender women compared to matched cisgender men and women [9, 18]. In the transgender women receiving oral estrogen, an increased risk for both VTE and IS was reported (hazard ratios for VTE and IS were 1.9 [95% CI, 1.0-3.5] and 2.4 [0.9-3.2] compared with cisgender men and 2.5 [1.4-4.5] and 3.2 [1.5-7.0] compared with cisgender women). No increased risk of VTE was reported for transgender women receiving nonoral estrogen (hazard ratios were 1.0 [0.4-2.4] vs cisgender men and 1.1 [0.4-2.6] vs cisgender women). However, the risk of IS was similarly elevated in groups of patients receiving oral or nonoral estrogen. The numbers of transgender women receiving transdermal or injectable estrogen were too low for a meaningful statistical comparison between risk of VTE or IS with different modes of estrogen administration in the transgender women. These initial results suggest that nonoral administration of E2 to transgender patients may be safer with respect to VTE but not IS. Larger studies will be needed to clarify these safety issues.

Our preliminary data indicate that SC administration of E2 has less of a first-pass hepatic effect than oral administration, specifically with respect to E2 conversion to E1. First, the weekly dose of E2 was much smaller when administered SC than when administered orally, which is consistent with less hepatic metabolism of E2. Second, in our subgroup of patients with serum E1 measured, levels in the oral group were markedly higher than in the SC group, consistent with previous studies comparing oral and transdermal administration [19, 20]. Thus, our results are consistent with less hepatic metabolism of E2 when administered SC compared to orally. Whether this difference in first-pass hepatic effects extends to other hepatic products such as clotting factors was not addressed in our study. To date the few studies evaluating effects of the elevations of E1 with oral E2 administration have not demonstrated a clinical advantage [20, 21]. On the other hand, some studies suggest increased risk with these elevations in E1 with respect to elevated biliary cholesterol saturation indices [22] and increased peak thrombin generation [10]. In addition, an in vitro study showed that E1 might have greater proinflammatory and protumorigenic activity of E1 than E2 [23]. In contrast to the difference in E2 metabolism by the liver with oral vs SC administration, no differences were demonstrated in effects on hepatic SHBG production.

Limitations of our study include a small sample size and retrospective study design. An additional limitation was the heterogeneity of the study population, as some individuals were receiving concomitant progestogens and/or spironolactone. A recent study has indicated that GAHT monotherapy with E2 is equally effective as E2 therapy with a progestogen and/or spironolactone for suppression of serum T [5]. A larger sample size, particularly for pre- and post-dose serum levels, would strengthen the power of these results. Additionally, the small number of patients in the study did not allow us to fully characterize safety. A larger prospective study would be helpful to assess broader aspects of safety such as risks of VTE and IS.

In conclusion, our data support that SC E2 at doses of 1.4 to 6 mg per week is a reasonable alternative to oral or IM E2 for GAHT for transgender women. SC E2 therapy may have the additional benefit of undergoing reduced first-pass metabolism when compared to oral therapy. These potential clinical benefits require additional investigation to determine whether they are present and to what degree.

## Disclosures

The authors have no conflicts of interest to disclose and received no funding for this study.

## Data Availability

Some or all datasets generated during and/or analyzed during the current study are not publicly available but are available from the corresponding author on reasonable request.

## Abbreviations

E1: estrone
E2: 17β-estradiol
GAHT: gender-affirming hormone therapy
GnRH: gonadotropin-releasing hormone
GnRHa: gonadotropin-releasing hormone agonist
IM: intramuscularly
IQR: interquartile range
IS: ischemic stroke
SC: subcutaneously administered
SHBG: sex hormone–binding globulin
TT: total testosterone
VTE: venous thromboembolism
